# MENTAL ILLNESS AND EMERGENCY DEPARTMENT USE IN VETERANS WITH DEMENTIA: DOES RESIDENCE MATTER?

**DOI:** 10.1093/geroni/igae098.2278

**Published:** 2024-12-31

**Authors:** Katherine Miller, Megan Shepherd-Banigan

**Affiliations:** Johns Hopkins University, Baltimore, Maryland, United States; Duke University Medical Center, Durham, North Carolina, United States

## Abstract

Likelihood of dementia increases among individuals with mental illness. For patients with Alzheimer’s-related and related dementia (ADRD), mental illness can complicate clinical care and may reduce care quality, leading to more intensive health service use and higher costs. For individuals living in nursing homes, this may be exacerbated as nursing home staff may not be trained to provide care appropriately to people with mental illness. We describe presence of mental illness (MI), defined as major depressive disorder, post-traumatic stress disorder, generalized anxiety disorder, bipolar disorder, or schizophrenia, in a cohort of Veterans with a new ADRD diagnosis stratified by nursing home residence versus community-dwelling. Of Veterans with new ADRD diagnoses, 96% are community-dwelling and 4% reside in nursing homes. Yet 19% of nursing home residents have pre-existing MI while 12% of community-dwellers have pre-existing MI. Among both community-dwellers and residents, those with pre-existing MI are younger at the time of ADRD diagnosis. In the year post-ADRD diagnosis, 17.5% and 9.5% of community-dwelling Veterans with and without pre-existing MI, respectively, had any emergency department (ED) visits. In contrast, 7.2% and 3.2% of nursing home residents with and without pre-existing MI, respectively, had any ED visits. Notably, a greater share of nursing home residents with pre-existing MI had any ED visits in the 2 years pre-ADRD diagnosis relative to community-dwellers with pre-existing MI (74% versus 43%, respectively). Our findings suggest that community-dwelling Veterans with pre-existing MI and newly diagnosed ADRD may benefit from targeted interventions to reduce ED use.

